# Inosine Pretreatment Attenuates LPS-Induced Lung Injury through Regulating the TLR4/MyD88/NF-κB Signaling Pathway In Vivo

**DOI:** 10.3390/nu14142830

**Published:** 2022-07-09

**Authors:** Bingyong Mao, Weiling Guo, Xin Tang, Qiuxiang Zhang, Bo Yang, Jianxin Zhao, Shumao Cui, Hao Zhang

**Affiliations:** 1 State Key Laboratory of Food Science and Technology, Jiangnan University, Wuxi 214122, China; maobingyong@jiangnan.edu.cn (B.M.); 7200112062@stu.jiangnan.edu.cn (W.G.); xintang@jiangnan.edu.cn (X.T.); zhangqx@jiangnan.edu.cn (Q.Z.); bo.yang@jiangnan.edu.cn (B.Y.); zhaojianxin@jiangnan.edu.cn (J.Z.); zhanghao61@jiangnan.edu.cn (H.Z.); 2 School of Food Science and Technology, Jiangnan University, Wuxi 214122, China; 3 National Engineering Research Center for Functional Food, Jiangnan University, Wuxi 214122, China

**Keywords:** inosine, lung injury, intestinal microbiota, TLR4/MyD88/NF-κB

## Abstract

Inosine is a type of purine nucleoside, which is considered to a physiological energy source, and exerts a widely range of anti-inflammatory efficacy. The TLR4/MyD88/NF-κB signaling pathway is essential for preventing host oxidative stresses and inflammation, and represents a promising target for host-directed strategies to improve some forms of disease-related inflammation. In the present study, the results showed that inosine pre-intervention significantly suppressed the pulmonary elevation of pro-inflammatory cytokines (including tumor necrosis factor-α (TNF-α) and interleukin-1β (IL-1β)), malondialdehyde (MDA), nitric oxide (NO), and reactive oxygen species (ROS) levels, and restored the pulmonary catalase (CAT), glutathione peroxidase (GSH-Px), superoxide dismutase (SOD), and myeloperoxidase (MPO) activities (*p* < 0.05) in lipopolysaccharide (LPS)-treated mice. Simultaneously, inosine pre-intervention shifted the composition of the intestinal microbiota by decreasing the ratio of Firmicutes/Bacteroidetes, elevating the relative abundance of Tenericutes and Deferribacteres. Moreover, inosine pretreatment affected the TLR4/MyD88/NF-κB signaling pathway in the pulmonary inflammatory response, and then regulated the expression of pulmonary iNOS, COX2, Nrf2, HO-1, TNF-α, IL-1β, and IL-6 levels. These findings suggest that oral administration of inosine pretreatment attenuates LPS-induced pulmonary inflammatory response by regulating the TLR4/MyD88/NF-κB signaling pathway, and ameliorates intestinal microbiota disorder.

## 1. Introduction

Lung injury is a life-threatening condition, and has attracted the wide attention due to its high morbidity and mortality [1,2]. Clinical evidence suggests that lung injury is generally accompanied by rapid alveolar injury, pulmonary infiltrates, uncontrolled inflammatory response, and excessive oxidative stress [3]. Among those, the abnormal inflammatory response plays a vital role in the process of lung injury, and is mainly represented by elevated concentrations of harmful reactive oxygen species (ROS) and insufficient cellular anti-inflammatory defenses [4]. High levels of ROS are frequently found in patients with lung injury, and can affect inflammatory signaling pathways—especially toll-like receptors/myeloid differentiation primary response 88/nuclear factor-kappa B (TLR4/MyD88/NF-κB) [5,6]. Activation of the TLR4/MyD88/NF-κB signaling pathway accelerates the recruitment of inflammatory cells, production of inflammatory molecules, and oxidative stresses, including tumor necrosis factor-α (TNF-α), interleukin-1β (IL-1β), interleukin-6 (IL-6), superoxide dismutase (SOD), and malondialdehyde (MDA) [7]. Thus, reducing ROS accumulation, inhibiting the TLR4/MyD88/NF-κB signaling pathway, and suppressing inflammatory cytokines can effectively prevent the process of lung injury.

Purine nucleosides are small molecules derived from the co-metabolism of intestinal microbiota, and possess an extensive range of physiological properties, such as anti-inflammatory, antioxidant and hepatoprotective effects [8,9]. Purine nucleosides are mainly composed of adenosine and its primary metabolite inosine. Recently, inosine has drawn wide attention because of its excellent anti-inflammatory effects. Mager et al. found that microbiome-derived inosine could elevate the effects of checkpoint blockade immunotherapy by activating antitumor T cells [10]. Our previous study showed that inosine pretreatment suppressed the inflammatory responses in the liver by regulating the TLR4/MyD88/NF-κB pathway [11]. In lung injury, regulatory T cells could prevent and/or treat related inflammation by inhibiting the secretion of IL-1β [12]. Therefore, whether inosine pretreatment can protect against acute lung injury, and whether it is related to the TLR4/MyD88/NF-κB pathway and IL-1β, needs to be further studied.

It is widely accepted that the intestinal microbiota plays a vital role in controlling the host energy metabolism and immune system [13], and dysbiosis of the intestinal microbiota can cause hyperglycemia, hyperlipidemia, intestinal barrier injury, and immune deficiency [14]. Liu et al. found that the decreases in the Firmicutes/Bacteroidetes ratio exhibited a noteworthy positive association with the levels of serum inflammatory cytokines, including IL-1β, IL-6, TNF-α, and TNF-β [15]. Moreover, some beneficial bacteria are negatively related to the inflammatory factors and oxidative stress levels, and positively related to the activity of antioxidant enzymes [16,17]. Therefore, whether the inflammatory state of lung injury can be altered by modulating the gut microbiota requires further investigation

To date, there are few studies concerning the potential mechanisms by which inosine ameliorates the lung injury induced by intraperitoneal injection of LPS. The purpose of this study was to assess the effects of inosine pretreatment on LPS-induced lung injury. More importantly, this study sought to explore whether inosine could change the composition of the intestinal microbiota, and to analyze the possible correlations between the intestinal microbiota and the lung-damage-associated parameters. The results should provide important knowledge to promote new strategies for the treatment of lung damage.

## 2. Materials and Methods

### 2.1. Materials

**Figure 1:**
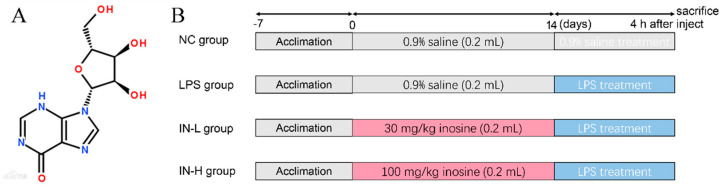
The structure of inosine (**A**); the experimental schedule (**B**).

Inosine from *Corynebacterium* spp. was obtained from Sangon Biotech Co., Ltd., (Shanghai, China), and its chemical structure is shown in Figure 1A. LPS was obtained from Sigma-Aldrich (Saint Louis, MO, USA). The assay kits for TNF-α, IL-1β, and IL-6 were obtained from R&D Systems (Shanghai, China). Malondialdehyde (MDA), superoxide dismutase (SOD), catalase (CAT), myeloperoxidase (MPO), lactate dehydrogenase (LDH), glutathione peroxidase (GSH-PX), and reactive oxygen species (ROS) assay kits were purchased from Jiancheng Bioengineering Institute (Nanjing, China).

### 2.2. Design of Animal Experiments

Forty male C57BL/6 mice (six weeks old, 19 ± 1 g) were provided by the Animal Research Center (Shanghai, China) and housed in controlled conditions. All mice were allowed free access to standard chow and water. After 1 week of adaptive feeding, all mice were randomly classified into 4 groups (*n* = 10) (Figure 1B). Mice in the control group (NC) and model group (LPS) were orally administrated with sterile NaCl solution (0.9%, *w*/*v*). Mice in the treatment groups—namely, the IN-L and IN-H groups—were orally administered with different doses of inosine (30 and 100 mg/kg/day, respectively). After inosine treatment for 14 days, mice in the NC group were intraperitoneally injected with sterile NaCl solution (0.9%, *w*/*v*). Mice in the other groups were intraperitoneally injected with the same volume of LPS solution (5 mg/kg). At 4 h after LPS injection, the feces were collected in sterile centrifuge tubes and stored at −80 °C, and then all mice were euthanized. The experimental protocol was approved by the Ethics Committee of Jiangnan University (No.20201115c0701230 [309]).

### 2.3. Biochemical Assays

A portion of the lung samples were homogenized with RIPA lysis buffer (Beyotime Biotechnology, Shanghai, China). The residues were removed by centrifugation (14,000× *g*, 10 min, 4 °C). The protein levels of the supernatant were detected using the BCA protein assay kit (Biotechnology, Shanghai, China). The pulmonary TNF-α, IL-1β, and IL-6 levels were detected using ELISA kits (R&D Systems, Shanghai, China). The pulmonary malondialdehyde (MDA), superoxide dismutase (SOD), catalase (CAT), myeloperoxidase (MPO), lactate dehydrogenase (LDH), glutathione peroxidase (GSH-Px), and nitric oxide (NO) levels were analyzed using commercial kits.

The reactive oxygen species (ROS) levels in the lung tissues were determined as described in a previous report [18]. In brief, the supernatants of the samples were diluted 200-fold in phosphate-buffered saline, and 0.1 mL of the diluted supernatant was mixed with 0.1 mL of DCFH-DA and transferred to 96-well plates. Then, the plates were placed in the control incubator for 5 min, and the fluorescence was quantified.

### 2.4. Lung Histopathology

Lung tissues were analyzed by hematoxylin and eosin (H&E) staining following a standard procedure described in a previous report [19]. In brief, the samples were collected, fixed in paraformaldehyde, and stained with H&E.

### 2.5. Intestinal Microbiota Analysis

The extraction of fecal DNA and amplification of the V3–V4 region of the 16S rRNA gene was implemented as described in a previous report [20]. The PCR products were purified using AMPure magnetic purification beads (Agencourt Brea, CA, USA) and then pooled into equal concentrations. A Qubit 2.0 Fluorometer (Thermo Fisher Scientific, Waltham, MA, USA) was applied to assess the quantity of sequencing libraries and sequenced on the Illumina MiSeq platform. The raw data from high-throughput sequencing were demultiplexed and quality-filtered using the QIIME2 platform. The results were assigned to operational taxonomic units (OTUs) by UCLUST, with a threshold of 97%.

The ACE, Chao1 and Shannon indices of intestinal microbiota were assessed on 15 June 2020 (https://www.microbiomeanalyst.ca/). Principal coordinates analysis (PCOA) based on Bray–Curtis dissimilarity was implemented using R software (v 4.1.2). In addition, Spearman’s correlation analysis was applied to analyze the relationships between intestinal microbiota and the key parameters related to inflammation and oxidative stress, using R software (v 4.1.2). On the basis of the Spearman analysis, heatmaps and networks were generated using TBtools software (Ver. 1.09861) and Cytoscape (v 3.6.2). Phylogenetic investigation of communities by reconstruction of unobserved states (PICRUSt2) was predicted using Xshell (v 7.0).

### 2.6. Quantitative RT-PCR

Total pulmonary RNA was collected using the commercial extraction kit (Carry Helix Biotechnologies Co., Ltd., Beijing, China). Then, total RNA was reversely transcribed using commercial kits (Takara, Dalian, China). qPCR was performed using a StepOnePlus Real-Time PCR System (Applied Biosystems, Foster City, CA, USA) with SYBR Premix Ex Taq II (Takara, Dalian, China). The pulmonary mRNA expression levels were normalized to the β-actin levels. The primers applied in the present study are presented in Table S1. The transcriptional levels of the genes related to inflammation and oxidative stress were computed by the 2^−ΔΔCt^ method.

### 2.7. Statistical Analysis

All data in the present study are presented as the means ± SEM, and were analyzed by one-way analysis of variance using GraphPad Prism 7.0 software (GraphPad, San Diego, CA, USA). The significance levels were set at *p* < 0.05 (significant) and *p* < 0.01 (extremely significant) relative to the LPS group.

## 3. Results

### 3.1. Inosine Pretreatment Alleviated LPS-Stimulated Inflammatory Response in Lung-Injured Mice

**Figure 2:**
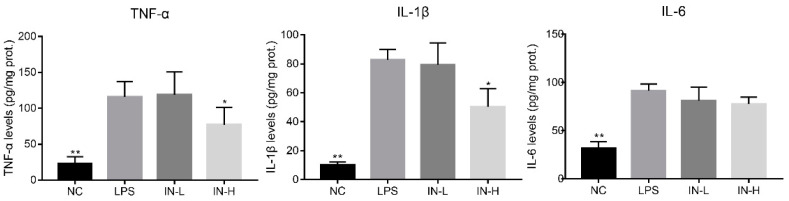
Effects of inosine pretreatment on the levels of TNF-α, IL-1β, and IL-6 in mice treated with LPS. * *p* < 0.05; ** *p* < 0.01, relative to the LPS group.

The anti-inflammatory role of inosine in LPS-treated mice was explored by detecting the concentrations of pro-inflammatory cytokines (i.e., TNF-α, IL-1β, and IL-6) in the lungs. As shown in Figure 2, the levels of colonic TNF-α, IL-6, and IL-1β were remarkably elevated in the LPS group compared with the NC group (*p* < 0.01). Pre-intervention with different concentrations of inosine had different effects on the pro-inflammatory cytokines—in particular, 100 mg/day inosine pre-intervention remarkably reduced the pulmonary TNF-α and IL-1β levels (*p *< 0.01). However, there was no statistical difference in the pulmonary IL-6 levels between the LPS and IN-H groups (*p* > 0.05).

### 3.2. Inosine Pretreatment Regulated LPS-Induced Oxidative Stress in Lung-Injured Mice

**Figure 3:**
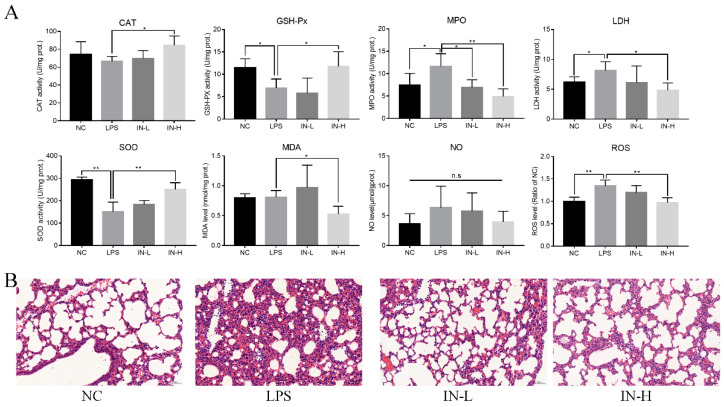
Effects of inosine pretreatment on the levels of CAT, GSH-Px, SOD, MPO, LDH, MDA, NO, and ROS in mice treated with LPS (**A**); histological examination of lung tissue by hematoxylin–eosin staining (**B**). * *p* < 0.05; ** *p* < 0.01, relative to the LPS group, n.s.: not significant.

Considering that oxidative stress is a major pathological characteristic of pulmonary inflammatory response in LPS-treated mice, the concentrations of MDA and NO in the lungs were measured to assess the amelioration caused by inosine pre-intervention (Figure 3A). There were no remarkable discrepancies in pulmonary MDA and NO levels between the NC group and the LPS group (*p* > 0.05), indicating that the LPS injection did not change the pulmonary MDA and NO levels within 4 h. However, inosine pre-intervention at 100 mg/kg significantly decreased the pulmonary MDA levels (*p* < 0.05). In addition, the pulmonary ROS levels were strongly associated with the progression of lung inflammation. LPS injection significantly elevated the pulmonary ROS levels relative to the NC group (*p* < 0.05), suggesting that the model of lung injury was successfully established. Inosine pre-intervention decreased the pulmonary ROS levels in a dose-dependent manner. Thus, the pre-intervention with inosine effectively improved LPS-induced oxidative stress in mice.

To understand the anti-inflammatory role of inosine in lung injury induced by LPS, pulmonary CAT, GSH-Px, SOD, MPO, and LDH activities were analyzed (Figure 3A). Relative to the NC group, the activity of pulmonary CAT was suppressed in the LPS group, although the discrepancy was not statistically remarkable (*p* > 0.05). Interestingly, oral administration of inosine at 100 mg/kg remarkably elevated the activity of pulmonary CAT compared with the LPS group (*p* < 0.05). In addition, the activities of pulmonary GSH-Px and SOD in the LPS group were remarkably inhibited relative to those of the NC group (*p* < 0.05), whereas inosine pre-intervention at 100 mg/kg recovered the activities of pulmonary GSH-Px and SOD (*p* < 0.05). LPS injection resulted in the elevation of the activities of pulmonary MPO and LDH. Nevertheless, inosine pre-intervention inhibited the activities of pulmonary MPO and LDH, especially at the dose of 100 mg/kg (*p* < 0.05). Thus, inosine ameliorated the pulmonary anti-inflammatory status in mice treated with LPS.

To further assess the amelioration effects of inosine in mice treated with LPS, the morphology of lung tissues in mice was observed using H&E staining (Figure 3B). Complete structural integrity was observed in the mice in the NC group. On the other hand, LPS treatment destroyed the pulmonary structure, increased the alveolar septa, accelerated the accumulation of red blood cells, and induced inflammatory cell infiltration and edema of the alveolar wall. Notably, inosine pre-intervention dramatically alleviated the inflammation and alveolar septa in both the IN-L and IN-H groups—especially the IN-H group.

### 3.3. Inosine Pretreatment Modulates the Intestinal Microbiota’s Structure

**Figure 4:**
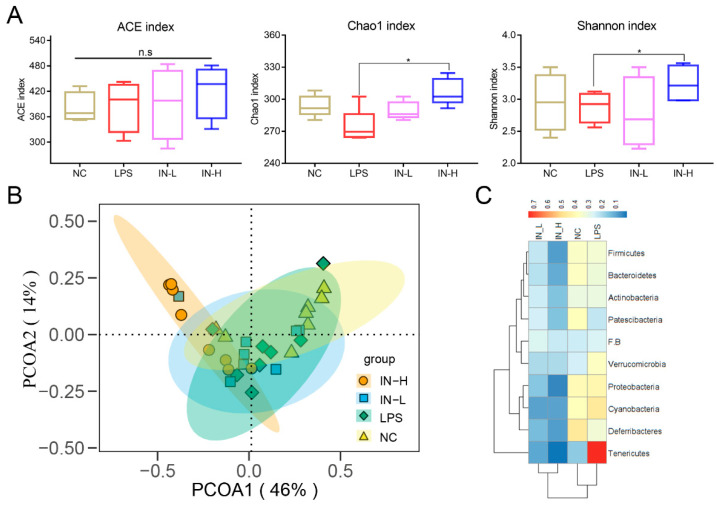
Effects of inosine on the alpha diversity and structure of the intestinal microbiota: Alpha diversity analysis including ACE index, Chao1 index, and Shannon index (**A**). PCA analysis of intestinal microbiota (**B**). Heatmap of proportions at the phylum level (**C**). * *p* < 0.05; relative to the LPS group, n.s.: not significant.

To investigate the effects of inosine on the intestinal microbiota, alpha diversity analysis—including ACE, Chao1, and Shannon indices—was applied to evaluate the richness and diversity of the intestinal microbiota. There were no remarkable discrepancies in the ACE, Chao1, or Shannon indices of the intestinal microbiota between the NC group and the LPS group (*p* > 0.05) (Figure 4A). However, the Chao1 and Shannon indices were remarkably elevated after pre-intervention with inosine. Subsequently, trends in the intestinal microbial communities between different groups were analyzed by PCOA (Figure 4B). In the four groups, the IN-L group had the most similar microbial community distribution to the control and model groups. Nevertheless, the IN-H group had the most discrepancy in microbial community compared to the other groups.

Subsequently, the microbial community composition at the phylum level was assessed (Figure 4C). The relative abundance of Verrucomicrobia and Tenericutes was significantly elevated in the LPS group relative to the NC group, while the relative abundance of Patescibacteria was decreased (Figure 4C). Interestingly, inosine pre-intervention significantly reduced the relative abundance of Actinobacteria, Bacteroidetes, Cyanobacteria, Deferribacteres, Firmicutes, Patescibacteria, Proteobacteria, Tenericutes, and Verrucomicrobia relative to the LPS group. Nevertheless, no differences in the composition of the intestinal microbiota were observed between the IN-L and IN-H groups.

At the genus level, there was a higher proportion of *GCA-900066225*, *Romboutsia*, *Tyzzerella 3*, *Rumen bacterium*, *Clostridia bacterium*, *Barnesiella* sp., *Streptococcus*, *GCA*-*900066575*, *Anaeroplasma*, *Lachnospiraceae UCG*-*001*, *Coprocossus 2*, *Family XIII UCG*-*001*, *Enterorhabdus*, and *Clostridiales* bacteria in the LPS group relative to the NC group, whereas inosine treatment clearly reversed those changes (Figure S1). Furthermore, the relative abundances of *Clostridium sensu stricto* 1, *Peptococcus*, *Bacillus*, *Family XIII AD3011 group*, and *Ruminococcaceae UCG*-*005* in the LPS group were lower than in the NC group, but inosine treatment did not significantly affect the trend of those genera. Thus, our data suggested that inosine pre-intervention attenuated the intestinal microbial disorders in LPS-treated mice.

### 3.4. PICRUSt2 Analysis

**Figure 5:**
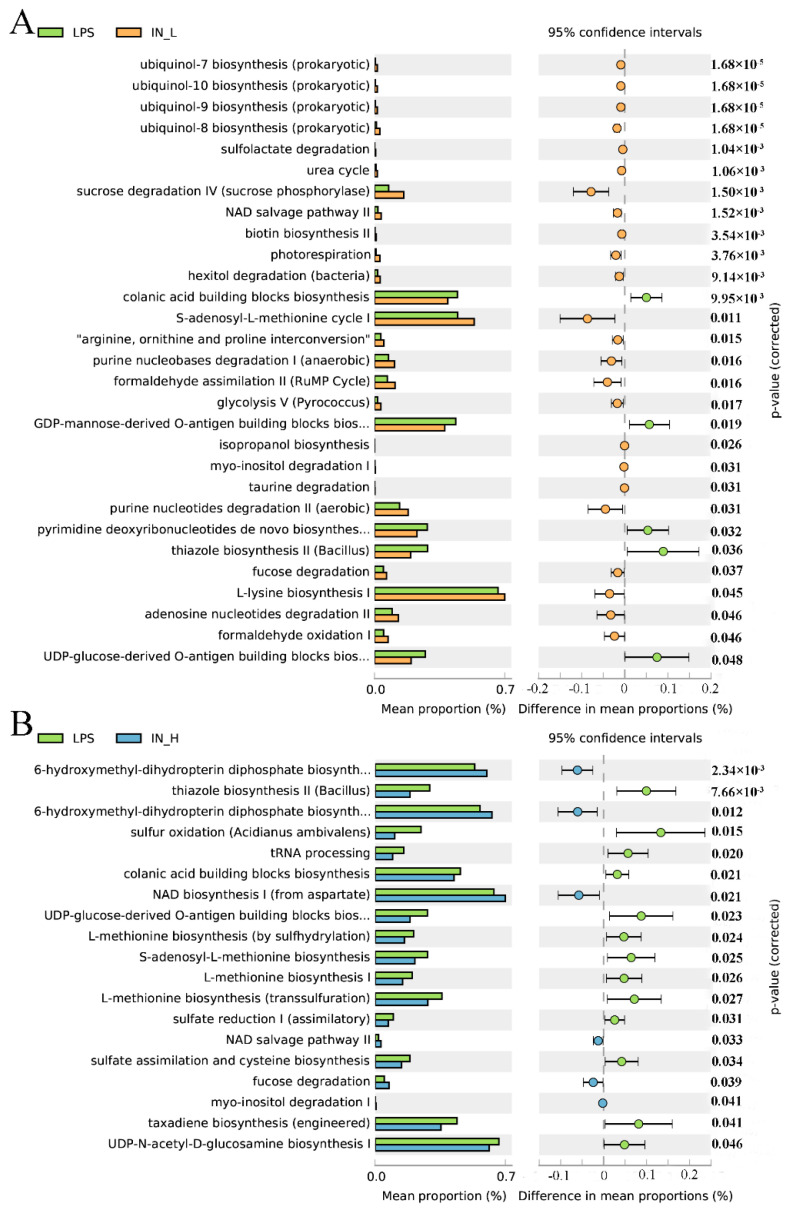
KEGG level 3 metabolic pathways: The remarkable differences in metabolic pathways between the LPS group and the IN-L group (**A**), and between the LPS group and the IN-L group (**B**).

PICRUSt2 was applied to investigate the bacterial functions of the members among different groups. A total of 22 potential functional profiles of intestinal microbiota were significantly altered between the NC and LPS groups (Figure S2), of which 5 potential functional profiles were remarkably upregulated and 17 potential functional profiles were remarkably downregulated in the LPS group relative to those in the NC group. Notably, 24 potential functional profiles were significantly increased and 5 potential functional profiles were remarkably reduced in the IN-L group compared to the LPS group (Figure 5A). However, 6 potential functional profiles were significantly increased and 13 potential functional profiles were remarkably decreased in the IN-H group compared with the LPS group (Figure 5B). There were remarkable discrepancies in the potential functional profiles of intestinal microbiota between the IN-L and IN-H groups, which may have been associated with the inosine-induced beneficial effects.

### 3.5. Associations between the Intestinal Microbiota and the Biochemical Indices Related to Lung Injury

**Figure 6:**
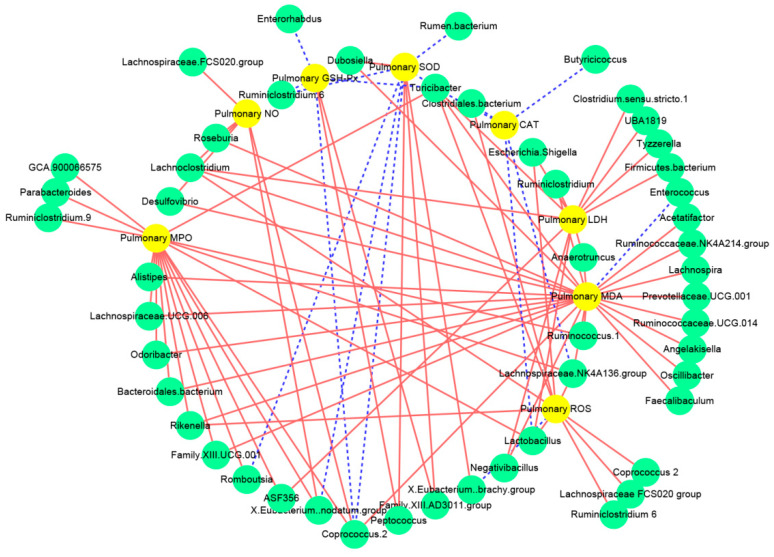
Correlation network established between intestinal microbiota and oxidative stress related to lung injury: The solid red line and dotted blue line represent the positive and negative correlation, respectively. In addition, the line width indicates the strength of correlation.

Pearson’s correlation analysis was applied to analyze the potential associations between the intestinal microbiota and the inflammatory indices related to lung injury (Figure 6 and Figure S3). Interestingly, the pulmonary ROS levels were positively associated with the abundance of *Rikenella*, *Lachnoclostridium*, *Lachnoclostridium* 2, *Lachnospiraceae FCS020 group*, and *Ruminiclostridium* 6. The pulmonary *SOD* activities were positively associated with the proportions of *Peptococcus*, *Family XIII AD3011 group*, *Eubacterium brachy group*, and *Negativibacillus*, and were negatively associated with the abundance of *Coprococcus* 2, *Eubacterium nodatum group*, *Romboutsia*, and *Ruminiclostridium* 6. Furthermore, the activity of pulmonary GSH-Px was positively related to the proportion of *Peptococcus* and *Family XIII AD3011 group*, but was negatively related to the abundance of *Coprococcus* 2 and *Enterorhabdus*.

### 3.6. Inosine Pretreatment Regulated the mRNA Expression of Genes Associated with Inflammation in Lung-Injured Mice

**Figure 7:**
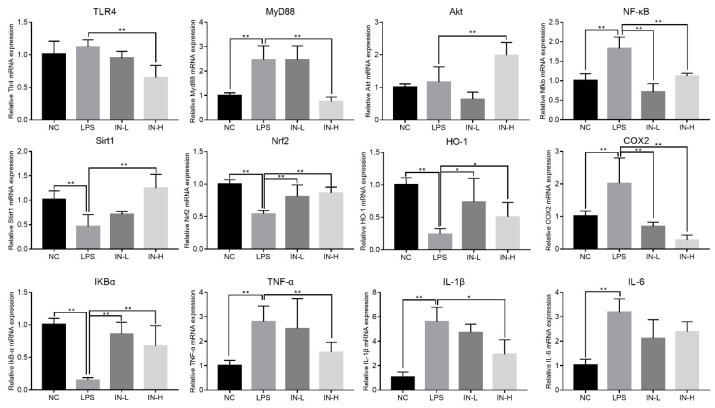
Impact of inosine pre-intervention on the transcription of genes related to the TLR4/MyD88/NF-κB signaling pathway in mice treated with LPS. * *p* < 0.05; ** *p* < 0.01, relative to the LPS group.

To explore the underlying mechanism of inosine in LPS-induced lung injury, the mRNA expression of genes related to inflammation was measured using RT-qPCR (Figure 7). Relative to the NC group, the transcription levels of MyD88, NF-κB, COX2, TNF-α, IL-1β, and IL-6 (but not TLR4) in the LPS group were dramatically upregulated (*p* < 0.05), but the transcription levels of Sirt1, Nrf2, HO-1, and IκBα in the LPS group were dramatically downregulated (*p* < 0.05). By contrast, pre-intervention with inosine dramatically reversed the changes in mRNA expression induced by LPS. In particular, inosine pre-intervention at 100 mg/kg remarkably downregulated the transcription levels of TLR4, MyD88, NF-κB, COX2, TNF-α, and IL-1β relative to the LPS group (*p* < 0.01), and significantly upregulated the transcription levels of Akt, Sirt1, Nrf2, HO-1, and IκBα (*p* < 0.05). These data suggest that inosine pre-intervention could improve LPS-induced lung injury by shifting the TLR4/MyD88/NF-κB signaling pathways.

## 4. Discussion

Lung injury is a complicated and lethal condition, which is universally characterized by higher levels of inflammation and abnormal oxidative stress status [21]. Therefore, the inhibition of inflammation accumulation is an essential strategy for preventing and/or treating lung injury. In the present study, inosine pre-intervention remarkably reduced the oxidative stress levels and elevated the activities of anti-inflammatory enzymes in LPS-treated mice. We also found that inosine pre-intervention significantly shifted the composition of the intestinal microbiota, and regulated the TLR4/MyD88/NF-κB signaling pathway.

Lung injury is regarded as associated closely with high concentrations of pro-inflammatory cytokines, especially TNF-α, IL-6, and IL-1β [22]. TNF-α is mainly secreted by macrophages and cell debris, and is a vital player in many inflammatory diseases, liver inflammation [23], arthritis [24], and colitis [25]. Higher levels of TNF-α were also discovered in mice with early pulmonary fibrosis, which is consistent with the results of this study [22]. In addition, a previous study found that the secretion of TNF-α is monitored by IL-1β [26]. IL-1β is a central monitor in the initiation of inflammation that is produced by infiltrating myeloid cells. Abnormal levels of IL-1β can cause serious local or systemic inflammatory reactions [27]. Furthermore, IL-6 is another common inflammatory cytokine that is produced by a variety of different cell types, and has frequently been found in metabolic inflammation and glycolipid metabolism disorder [28]. The above results indicate that inosine pre-intervention at 100 mg/kg·day can effectively attenuate LPS-induced lung injury through suppressing the pulmonary pro-inflammatory cytokines (i.e., TNF-α, IL-1β, and IL-6).

Furthermore, oxidative stress has been recognized as a pivotal pathophysiological characteristic of lung injury. The abnormal status of oxidative stress can accelerate the development of lung injury, and can even induce the beginning of lung cancer. It is well known that LPS-treated mice show abnormal oxidative stress status—such as increases in the levels of MDA and NO in the lungs—which is consistent with our results [29]. MDA served as the end product of polyunsaturated fatty acid peroxidation, which could reflect the level of host oxidative stress to a certain extent. High concentrations of MDA damage the integrity of cells through the crosslinking and polymerization of proteins or DNA. In addition, NO is produced from arginine by nitric oxide synthase, and is widely scattered in the organism. NO is generally considered to be a marker of inflammation in the lungs because it can facilitate the secretion of pro-inflammatory cytokines [30]. Therefore, reducing the production of MDA and NO can be an effective measure to ameliorate lung inflammation. Interestingly, inosine pre-intervention minimized the increase in MDA and NO caused by LPS. In addition, the activities of MPO, LDH, CAT, GSH-Px, and SOD were measured in this study, in order to estimate the influence of inosine on antioxidant and anti-inflammatory properties in LPS-treated mice. MPO serves as a biomarker of neutrophils, as its concentration was positively associated with the amounts of neutrophils in tissues. LDH mainly exists in the cytoplasm after cell death and local inflammation of cells, and high activity of LDH is regarded as a biomarker of lung injury. Our results showed that inosine pre-intervention reduced the pulmonary MPO and LDH levels, indicating that inosine directly protected the lungs against LPS toxicity, and prevented the development of lung injury. In addition, LPS treatment can partially induce lung injury by inhibiting the antioxidant enzymes (such as SOD, GSH-Px, and CAT) [31]. The superoxide radicals can be transformed by SOD into hydrogen peroxide, which is then degraded to water and oxygen by GSH-Px and CAT [31]. Therefore, increases in SOD, GSH-Px, and CAT activities are important strategies to protect the cells from oxidative stress. We found that inosine pre-intervention elevated the activities of pulmonary SOD, GSH-Px, and CAT, suggesting that the protective effects of inosine on LPS-induced lung injury may be attributed to the elevated levels of antioxidant enzymes.

ROS represent a wide class of molecules that play an essential role in the signaling pathways associated with inflammatory response. In this study, intraperitoneal injection of LPS induced increases in pulmonary ROS levels, which is consistent with previous findings [32]. ROS can easily destroy the structure of cells and the function of organs through changing the mitochondrial respiratory chain enzymes, lipid peroxidation, and modifications of membrane transport proteins [33]. In addition, overproduction of ROS subjects a biosystem to oxidative stress, resulting in the synthesis, secretion, and accumulation of pro-inflammatory cytokines [34]. Thus, the reduction of ROS is a vital measure for effectively maintaining cellular homeostasis. Our data showed that inosine pre-intervention remarkably reduced the pulmonary ROS levels, indicating that inosine may be a promising anti-inflammatory substance for relieving/treating lung damage.

The intestinal microbiota has been attracting a lot of attention because its composition is strongly associated with the host’s physiology. Furthermore, increasing research has suggested that the increasing usage of active substances induces alterations of the intestinal microbiota, which can influence the host immune response and energy metabolism [35]. Firmicutes and Bacteroidetes are the two dominant phyla in the intestinal tract, accounting for more than 80%. Thus, the balance of Firmicutes and Bacteroidetes is extremely important to maintain human health. Jia et al. found that the reduction in Firmicutes/Bacteroidetes ratio may contribute to mitigating the host’s systemic low-grade inflammation [36]. Moreover, patients with inflammatory bowel disease also exhibited increases in the Firmicutes/Bacteroidetes ratio, supporting the emerging view that the Firmicutes/Bacteroidetes ratio is closely associated with host inflammation [37]. Our results showed that inosine pre-intervention slightly reduced the Firmicutes/Bacteroidetes ratio, which may be beneficial for relieving the development of lung inflammation. In addition, patients with inflammatory bowel disease presented a higher abundance of *Tenericutes* when compared with healthy people [38]. *Deferribacteres* play a vital role in maintaining the bowel’s iron balance, and their abundance is positively correlated with the risk of diseases [39]. In this study, we also found that inosine pre-intervention reduced the relative abundance of *Tenericutes* and *Deferribacteres*. Therefore, we speculate that inosine improved host immunity partially by altering the intestinal microbiota structure. At the genus level, *Candidatus arthromitus* plays a vital role in promoting the immune system maturation [40]. *Anaerostipes* acts as one of the most important probiotics, is positively associated with L-glutamine, riboflavin B2, and IL-10 levels, and may elevate the beneficial effects on the cardiac functions of the host [41]. *Anaeroplasma*, belonging to Mollicutes class, is an opportunistic pathogen that can stimulate a series of immune responses [42]. *Turicibacter* has been described as being responsible for the process of intestinal inflammation [43]. As expected, inosine pre-intervention remarkably elevated the proportions of *Candidatus arthromitus* and *Anaerostipes*, and reduced the proportions of *Anaeroplasma* and *Turicibacter*. In addition, a previous report suggested that the abundance of *Akkermansia* and *Bifidobacterium* is positively associated with the intestinal inosine levels [10]. In the present study, inosine pretreatment slightly reduced the relative abundance of *Akkermansia* and *Bifidobacterium* in LPS-treated mice.

To deeply explore the underlying mechanisms of the ameliorating influence of inosine pre-intervention on lung injury induced by LPS, the transcription levels of genes related to inflammatory response were analyzed. The TLR4/NF-κB/IκBα signaling pathway is generally considered to be the typical signaling pathway related to the production of immunomodulators by activating macrophages [44]. TLR4 serves as the specific receptor of LPS originating from Gram-negative bacteria, and is mainly distributed in monocytes, dendritic cells, and macrophages. Overexpression of TLR4 stimulates the intracellular signaling pathways associated with lung injury, including the upregulation of MyD88 and NF-κB transcription [45]. However, the activation of MyD88 could inhibit the transcription levels of IκBα, and then upregulate the transcription levels of NF-κB. Some reports showed that NF-κB contributes to impairing the structure of cells and tissues by elevating the secretion of some inflammatory factors [46]. Our data showed that the expression of TNF-α, IL-1β, and IL-6 was dramatically upregulated in the LPS group as compared with the NC group. These inflammatory cytokines play a vital role in the beginning and development of inflammation in LPS-induced lung injury [47]. TNF-α is an outstanding regulator that induces pneumocyte apoptosis. A previous study showed that TNF-α production is one of the early stages of various types of inflammatory diseases, especially for lung injury [48]. IL-1β is involved in the inflammatory and immune responses, promoting neutrophil recruitment and activating the site of inflammation [49]. In addition, IL-6 has been strongly correlated with inflammatory disease, and elevates NF-κB activation in the histiocytes [50]. Moreover, our results showed that LPS treatment could inhibit the transcription of Sirt1, Nrf2, and HO-1, and elevated the transcription of COX2, which is consistent with the findings of Han et al. [51]. Sirt1 serves as an immunity regulator involved in many inflammation-associated diseases. Sirt1 activation can control inflammatory responses by suppressing the transcription of NF-κB [52]. In addition, overexpression of Sirt1 can elevate the HO-1 expression and the activity of GSH-Px and CAT by stimulating Nrf2 expression [53]. HO-1 is an endogenous antioxidant enzyme whose transcription is regulated by Nrf2 [54]. HO-1 plays a vital role in maintaining the homeostasis of organelles in cells, and prevents the cell damage induced by inflammation and oxidative stress by ameliorating mitochondrial dynamics [55]. Therefore, inosine pre-intervention can ameliorate lung injury by altering the TLR4/MyD88/NF-κB signaling.

## 5. Conclusions

In the present study, we found that inosine pre-intervention ameliorated LPS-induced lung injury by suppressing the secretion of pro-inflammatory cytokines, inhibiting oxidative stress and pulmonary ROS levels, and regulating TLR4/MyD88/NF-κB signaling. In addition, inosine pre-intervention shifted the structure of the intestinal microbiota—especially the increases in the proportions of *Tenericutes* and *Deferribacteres*. These results are conducive to further understanding of the mechanisms of inosine pre-intervention to alleviate lung injury by altering the TLR4/MyD88/NF-κB signaling pathway and the composition of the gut microbiota, and can hence guide further development of inosine-related commodities.

## Data Availability

Not applicable.

## Supplementary Materials

The following supporting information can be downloaded at: https://www.mdpi.com/article/10.3390/nu14142830/s1, Figure S1: Heatmap of relative abundance at the genus level; Figure S2: The remarkable differences in metabolic pathways between the LPS group and the IN-L group; Figure S3: Heatmap of Spearman’s correlations between the key intestinal microbial phylotypes and the parameters related to acute liver injury and inflammation; Table S1: Primer sequences for quantitative real-time PCR of hepatic genes.
