# Supplementary material for: Inosine Pretreatment Attenuates LPS-Induced Lung Injury through Regulating the TLR4/MyD88/NF-κB Signaling Pathway In Vivo
Source: Nutrients. 2022 Jul 9;14(14):2830. doi: 10.3390/nu14142830 (PMC9318366; doi:10.3390/nu14142830)
Supplement: Supplementary file 1 [file nutrients-14-02830-s001.zip › nutrients-1795090-supplementary.pdf]

Supplementary materials

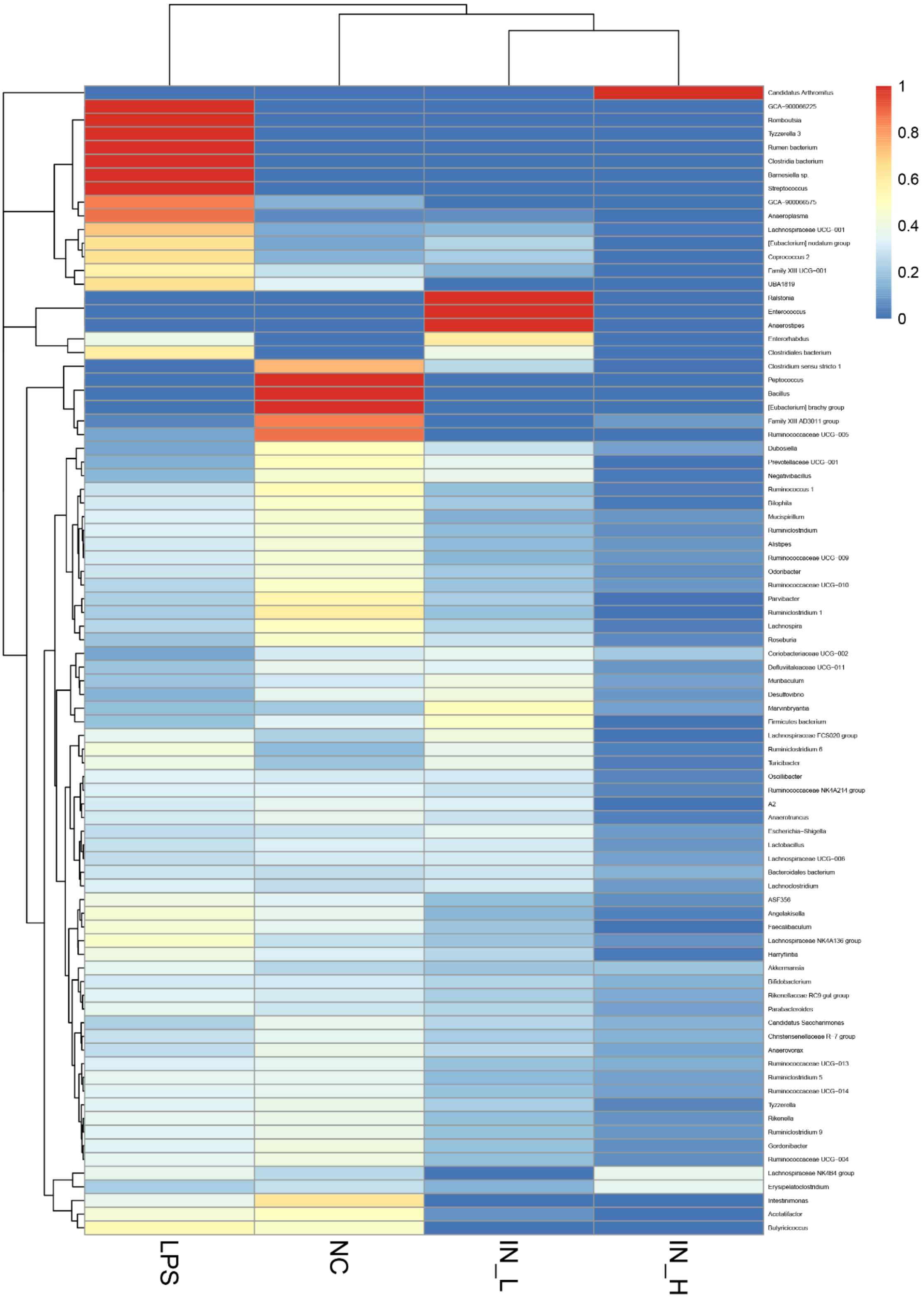

Figure S1 Heatmap of relative abundance at genus level.

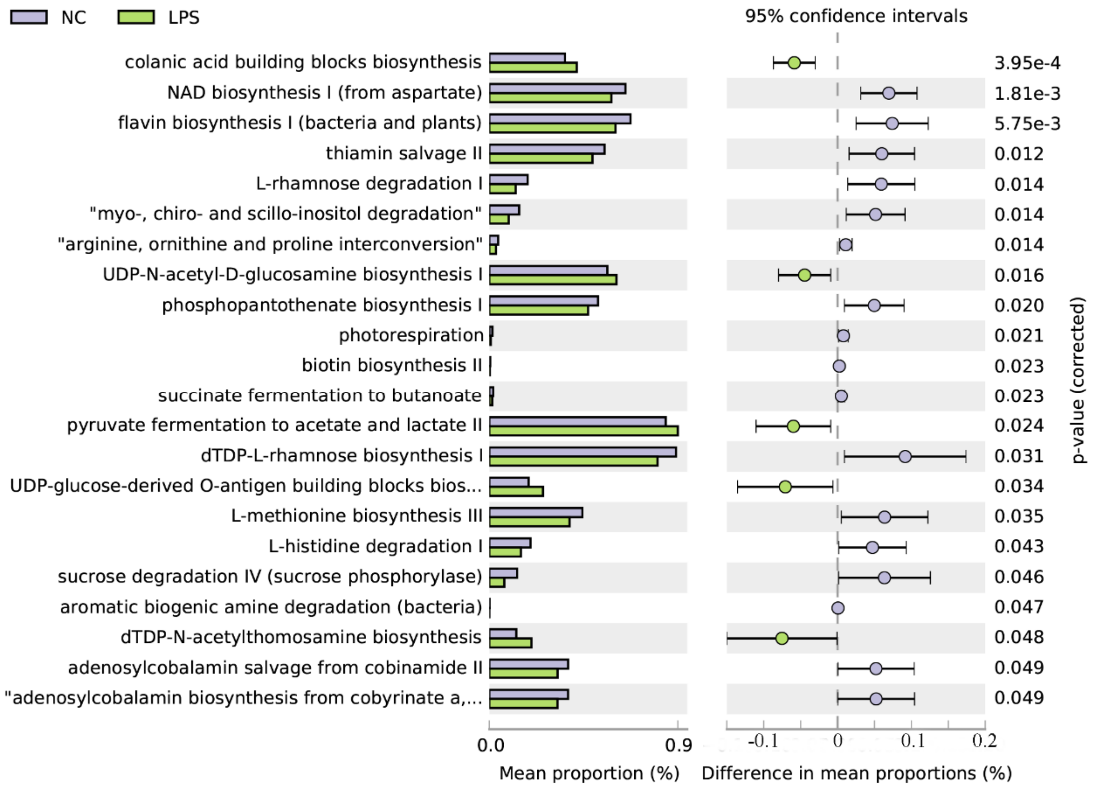

Figure S2 The remarkably differences of metabolic pathways between the LPS group and the IN-L group

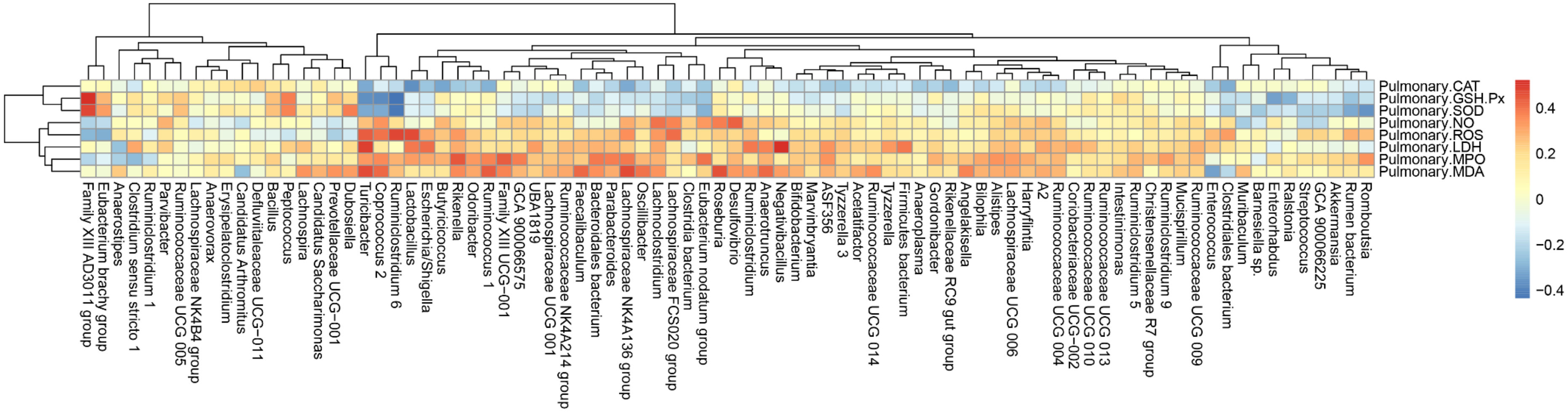

Figure S3 Heatmap of Spearman's correlations between the key intestinal microbial phylotypes and the parameters related to acute liver injury and inflammation.

Table S1. Primer sequences for quantitative real-time PCR of hepatic genes.

Primers	Forward Sequences (5′→3′)	Reverse Sequences (5′→3′)
TLR4	GGCAGCAGGTGGAATTGTAT	AGGCCCCAGAGTTTTGTTCT
MyD88	GTGCCGTCGGATGGTAGTG	GACAGTGATGAACCGCAGGAT
Akt	TCTACAACCAGGACCACGAGA	CTCCATGAGGATCAGCTCGAAC
Sirt1	AGAACCACCAAAGCGGAAA	TCCCACAGGAGACAGAAACC
Nfr2	CCTCCGCTGCCATCAGTCAGT	TCGGCTGGGACTCGTGTTCA
IKBα	ACCAACCAGCCAGAAATCG	TCACAGGCAAGGTGTAGAGGG
NF-κB	CGCCCCTTATCGACCACC	CCTTCTCCCAAGAGTCGTCCA
COX2	GGGAGTCTGGAACATTGTGAA	GCACGTTGATTGTAGGTGGACTGT
HO-1	ACATCCAAGCCGAGAATGCTG	CCAGTGAGGCCCATACCAGA
TNF-α	TCTTCTCATTCCTGCTTGTGGC	ACTTGGTGGTTTGCTACGACG
IL-1β	TCAAATCTCGCAGCAGCACATC	CGTCACACACCAGCAGGTTATC
IL-6	CCCCAATTTCCAATGCTCTCC	CGCACTAGGTTTGCCGAGTA
